# Sea stars generate downforce to stay attached to surfaces

**DOI:** 10.1038/s41598-021-83961-z

**Published:** 2021-02-25

**Authors:** Mark Hermes, Mitul Luhar

**Affiliations:** University of Southern California, Aerospace and Mechanical Engineering, Los Angeles, 90089 USA

**Keywords:** Mechanical engineering, Fluid dynamics

## Abstract

Intertidal sea stars often function in environments with extreme hydrodynamic loads that can compromise their ability to remain attached to surfaces. While behavioral responses such as burrowing into sand or sheltering in rock crevices can help minimize hydrodynamic loads, previous work shows that sea stars also alter body shape in response to flow conditions. This morphological plasticity suggests that sea star body shape may play an important hydrodynamic role. In this study, we measured the fluid forces acting on surface-mounted sea star and spherical dome models in water channel tests. All sea star models created downforce, i.e., the fluid pushed the body towards the surface. In contrast, the spherical dome generated lift. We also used Particle Image Velocimetry (PIV) to measure the midplane flow field around the models. Control volume analyses based on the PIV data show that downforce arises because the sea star bodies serve as ramps that divert fluid away from the surface. These observations are further rationalized using force predictions and flow visualizations from numerical simulations. The discovery of downforce generation could explain why sea stars are shaped as they are: the pentaradial geometry aids attachment to surfaces in the presence of high hydrodynamic loads.

## Introduction

Intertidal sea stars often function in environments with extreme hydrodynamic loads^1^ that can compromise their ability to remain attached to and move on surfaces^2^. While behavioral responses such as burrowing into sand or sheltering in rock crevices can help minimize hydrodynamic loads, previous work shows that sea stars also alter body shape in response to flow conditions^3^. This morphological plasticity suggests that sea star body shape and size may play an important hydrodynamic role. Specifically, Hayne and Palmer^3^ demonstrated that the arms of the purple sea star (*Pisaster ochraceous*) narrow and lose mass when transplanted to a more wave-exposed environment. The authors hypothesized that this transformation is a functional response to wave intensity: that by changing shape they are minimizing drag or related hydrodynamic forcing. Further, Computational Fluid Dynamics (CFD) simulations of wave-exposed sea star models showed a decrease in both lift and drag coefficients compared to models of sheltered sea stars, suggesting that the observed shape adaptation conferred a hydrodynamic benefit.

In this paper, we evaluate the hydrodynamics associated with sea star body shapes in turbulent flow conditions via laboratory experiments and CFD simulations. Though natural sea stars can exhibit significant diversity in surface texture, number of arms, and other morphological properties, we limit our study to smooth pentaradial shapes to isolate the effect of arm aspect ratio. Specifically, we consider models of comparable shape and size to adult purple sea stars. Sea stars are known to increase dramatically in size from the early juvenile to the adult stage^4,5^. The evolution in the hydrodynamic response during this development is outside the scope of the present study. In addition to providing insight into previous biological observations, the present effort has potential applications in fields ranging from flow control to shape optimization for vehicles.

Existing research investigating flow over surface-mounted objects has focused on characterizing the effect of surface fouling, designing drag-reducing structures, and studying airflow around buildings^6–9^. A variety of geometries have been considered, including cubes^10,11^, circular cylinders^12^, hemispheres^12–14^, pyramids^15,16^, cones^17–19^, and triangular cylinders^20,21^. Most of these studies provide drag and drag-coefficient estimates. Measurements of lift are rarer, in part because lift forces may be less important for the applications described. However, lift is a useful performance metric for cases in which surface attachment is a concern.

Perhaps the closest shapes to the sea star that have been studied are pyramids and triangular cylinders. Measurements made by Ikhwan^15^ indicate that pyramids generate lift forces that increase with increasing aspect ratio. On the other hand, Iungo and Baresti^20^ show that triangular cylinders generate downforce. This downforce is shown to originate from an upward deflection of the wake behind the cylinders, and its magnitude increases with increasing steepness of the triangular cross-section.

The experiments performed in this paper similarly show downforce generation for sea star models that is associated with an upward deflection of fluid flow around the body. However, downforce is not observed in experiments with spherical domes of comparable size to the sea star models. These observations, together with results from complementary CFD simulations of flow over cones, pyramids, and triangular cylinders, suggest that the radially symmetric sea star geometry generates a hydrodynamic response similar to (nearly) two-dimensional triangular cylinders.

## Results

We mounted 3-D printed sea star and spherical dome models to a load cell in a water channel to study the effect of morphology on the mean drag and lift forces ($F_d$, $F_L$) generated by these objects across a range of flow speeds (*U*). The effect of sea star morphology was studied by varying the arm aspect ratio *AR* over the range of values reported for *Pisaster ochraceous*^3^; here, *AR* is defined as the ratio between the arm length measured from the distal tip to the central axis and the arm width measured at the base intersection. We also measured the effect of body orientation with respect to the flow, $\Theta$, for the pentaradial sea star models using a servo motor positioning system. We supplemented these force measurements with PIV-based flow visualization and control volume analyses. To provide additional qualitative and quantitative insight into the experimental observations, we also pursued CFD simulations for a limited range of geometries. Details regarding model design, experimental apparatus, and analysis methods can be found in Sect. 4.

### Effect of aspect ratio and orientation on drag and lift

**Figure 1:**
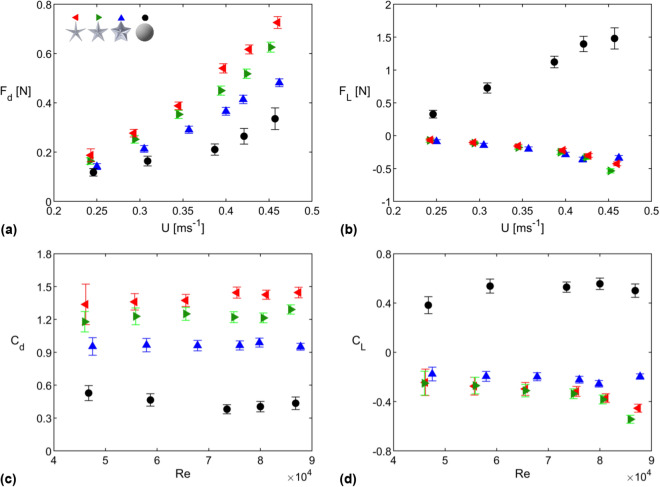
Mean (**a**) drag force, (**b**) lift force, (**c**) drag coefficient, and (**d**) lift coefficient values for sea star and spherical dome models shown as functions of freestream velocity (**a**,**b**) and Reynolds number (**c**,**d**). The sea star models have aspect ratios $AR = 4.0$ (red symbols), 2.5 (green symbols), and 1.5 (blue symbols).

Figure 1 compares the mean drag and lift generated by three sea star models of varying aspect ratio against the drag and lift generated by a spherical dome of similar height and base diameter as the sea star models. For these measurements, the sea star models were oriented at $\Theta = 0^\circ$, i.e., with one limb pointing into the oncoming flow. As expected, the magnitude of the drag and lift forces generated by the models increases with increasing freestream velocity. However, the corresponding drag and lift coefficients ($C_d$, $C_L$; see Eqs. (2) and (3)) show more limited variation with Reynolds number (*Re*, defined using base diameter; see Eq.(4)). Perhaps the most striking feature of the results presented in Fig. 1 is that all sea star models generate downforce (i.e., $F_L<0$ and $C_L < 0$) while the spherical dome model generates positive lift forces that are much higher in magnitude. Lift coefficient values for all three sea star models are similar within uncertainty at the lowest Reynolds number. However, measurements made at higher Reynolds numbers suggest that the $AR = 1.5$ sea star model (blue symbols) generates the least downforce and has the lowest $|C_L|$. Importantly, though the pentaradial sea star models generate downforce, they also incur a drag penalty compared to the spherical dome. The drag coefficients for the sea star models ($C_d > 0.9$) are significantly higher than those for the spherical dome ($C_d < 0.6$). Further, the drag coefficients for the sea star models increase with increasing aspect ratio.

**Figure 2:**
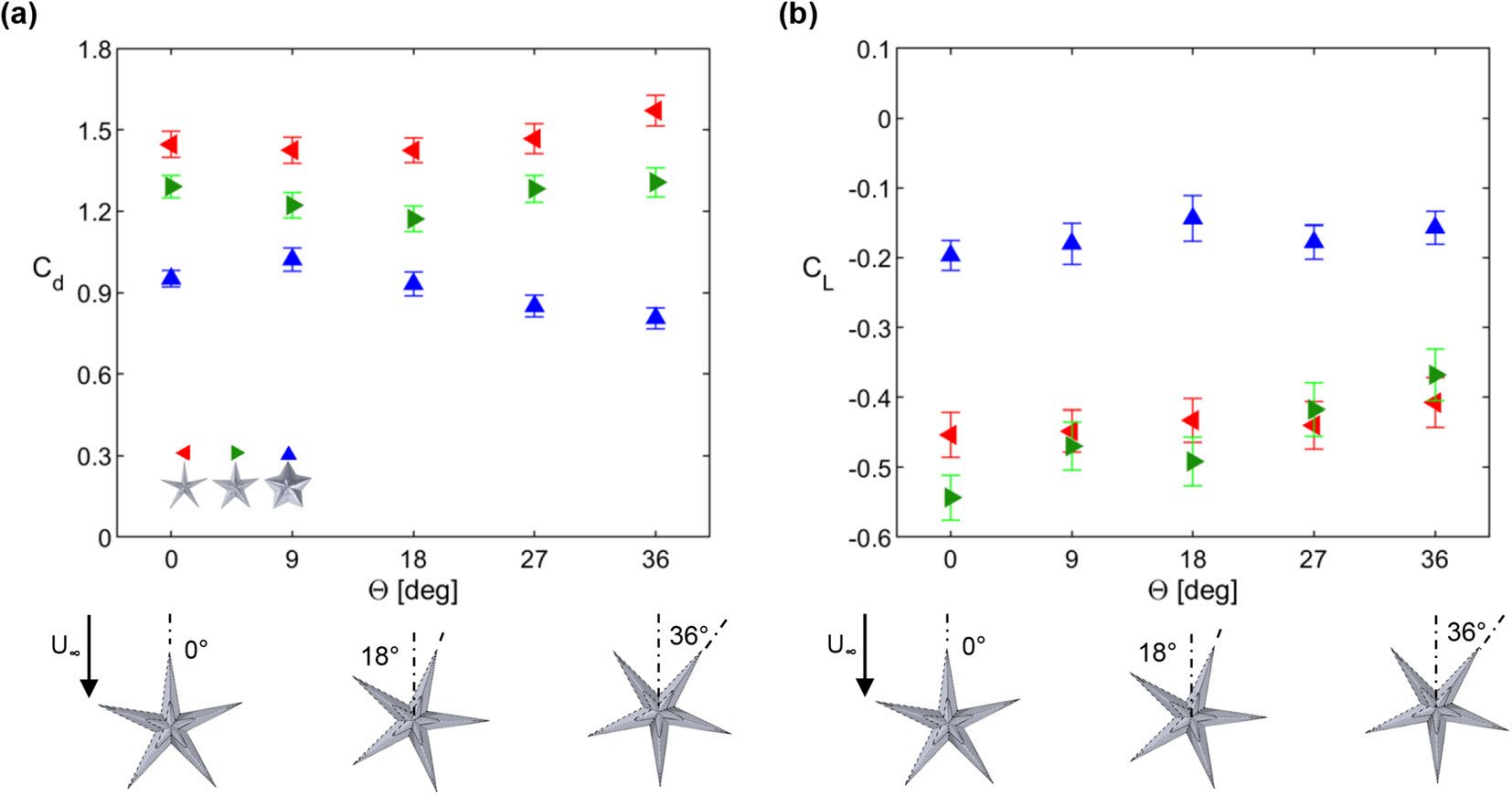
Drag and lift coefficient values for sea star models for varying orientation angles at flow speed $U \approx 0.46$ ms$^{-1}$. Given the pentaradial symmetry of the sea star models, $C_d$ and $C_L$ for $\Theta = 36\,^\circ$ to $\Theta = 72\,^\circ$ can be estimated by mirroring the data shown in this figure.

Figure 2 shows drag and lift coefficients for all three sea star models as a function of the orientation angle $\Theta$ for the highest Reynolds number case shown in Fig. 1. Consistent with the results from Fig. 1, there is a monotonic increase in $C_d$ as a function of aspect ratio. However, the drag coefficient values show no consistent trend with respect to orientation. Lift coefficients for all three geometries similarly show no clear trend with respect to orientation, though there is a consistent increase in $C_L$ with $\Theta$ for the model with $AR = 2.5$ (green symbols). In general, measured $C_L$ values for the models with $AR = 4.0$ (red symbols) and $AR = 2.5$ (green symbols) are significantly lower than the values measured for $AR=1.5$ (blue symbols). Together, these observations indicate that the drag and lift forces generated by the sea star models are relatively insensitive to orientation, and confirm that $C_d$ and $|C_L|$ increase with increasing *AR*.

### PIV flow visualization and control volume analysis

**Figure 3:**
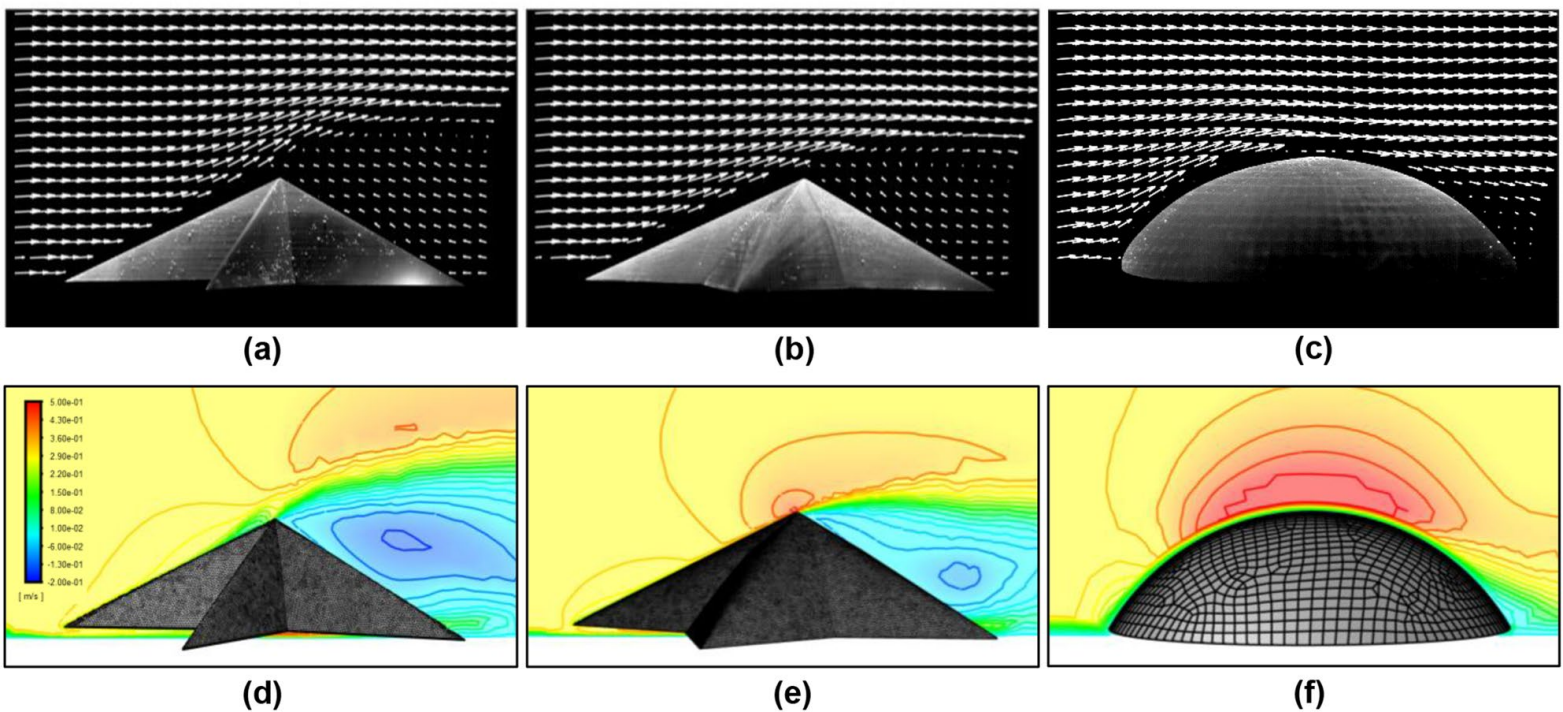
Mean flow visualization from experiments (**a**–**c**) and CFD simulations (**d**–**f**) for: $AR = 4.0$ sea star model (**a**,**c**); $AR = 1.5$ sea star model (**b**,**e**); and spherical dome (**c**,**f**). The experiments show results for $U = 0.47 \pm 0.01$ ms$^{-1}$ while the simulations were performed for $U = 0.35$ ms$^{-1}$. Panels (**a**–**c**) show the vector field estimated from PIV while panels (**d**–**f**) show contours of the mean velocity in the streamwise direction. All panels show the flow field at the central (or median) plane of the models. The sea star models are oriented at $\Theta = 0^\circ$. Figure created using Ansys Fluent 2019 R2 https://www.ansys.com/.

To provide further insight into the force measurements shown in Fig. 1, we pursued PIV experiments at freestream velocity $U = 0.47 \pm 0.01$. Though the fluid forces acting on the objects arise from three-dimensional flow fields, the planar mean flow visualizations shown in Fig. 3a–c provide a partial physical explanation for the observed trends in drag and lift. Specifically, the sharp apex creates a distinct separation point for the flow over the sea star models. In contrast, the flow over the spherical dome has a separation point much further down the body, resulting in a smaller wake region compared to the sea star models. Further, the wake behind the high aspect ratio sea star model is larger than the wake behind the low aspect ratio sea star model. These observations are qualitatively consistent with the drag force measurements shown in Fig. 1a: the sea star models generate more drag than the spherical dome, and the drag force generated increases with increasing aspect ratio. Importantly, the wakes behind the sea star models clearly show an upward redirection of the flow beyond the apex. An upward redirection of the flow is not observed for the spherical dome due to the delayed separation. These observations provide a qualitative explanation for the lift trends observed in Fig. 1b. The downforce experienced by the sea star models is a consequence of the upward redirection of fluid momentum in the wake.

**Table 1:** Mean values for lift ($F_L{^\prime }$) and drag ($F_d{^\prime }$) per unit length estimated from control volume analyses of planar vector fields obtained from PIV.

Model	$F_L{^\prime }$ [Nm$^{-1}$]	$F_d{^\prime }$ [Nm$^{-1}$]
Sea star, $AR = 4.0$	− 1.32	5.94
Sea star, $AR = 1.5$	− 1.19	4.06
Spherical dome	2.41	3.27

We also used a control volume approach (described in Sect. 4) to estimate drag and lift forces per unit length ($F_d{^\prime }$, $F_L{^\prime }$) from the planar PIV measurements. Figure 1 lists the mean values for $F_d{^\prime }$ and $F_L{^\prime }$ obtained after averaging over all PIV frames.

Consistent with the load cell measurements of lift shown in Fig. 1b, $F_L{^\prime }$ is positive for the spherical dome and negative for the two sea star models. In addition, the magnitude of the estimated lift per unit length ($|F_L{^\prime }|$) for the spherical dome is nearly twice that for the sea star models. Both sea star models have similar $F_L{^\prime }$ values though the $AR=4.0$ model is estimated to generate slightly higher downforce per unit length. Similarly, the estimates for $F_d{^\prime }$ are also consistent with the drag measurements shown in Fig. 1a. The high aspect ratio sea star model has higher $F_d{^\prime }$ than the low aspect ratio model, and the low aspect ratio star has higher $F_d{^\prime }$ than the spherical dome.

### CFD simulation results

To supplement the experiments, we pursued CFD simulations in ANSYS Fluent for a subset of the sea star models, the spherical dome, and several related geometries. The additional shapes (hemisphere, cone, pyramid, triangular prism; see Fig. 5) were created to have the same frontal area and height as the sea star. For the triangular prism, the streamwise length was set to be similar to the base width of the sea star model, $L = 19$ cm, such that the prism cross-section was identical to the midplane cross-section of the sea star model shown in Fig. 3a. These shapes were tested in water flow with an inlet speed of $U = 0.35$ ms$^{-1}$. Experimental data for the sea star and spherical dome models shown in Fig. 1 were linearly interpolated for comparison at this flow speed.

**Figure 4:**
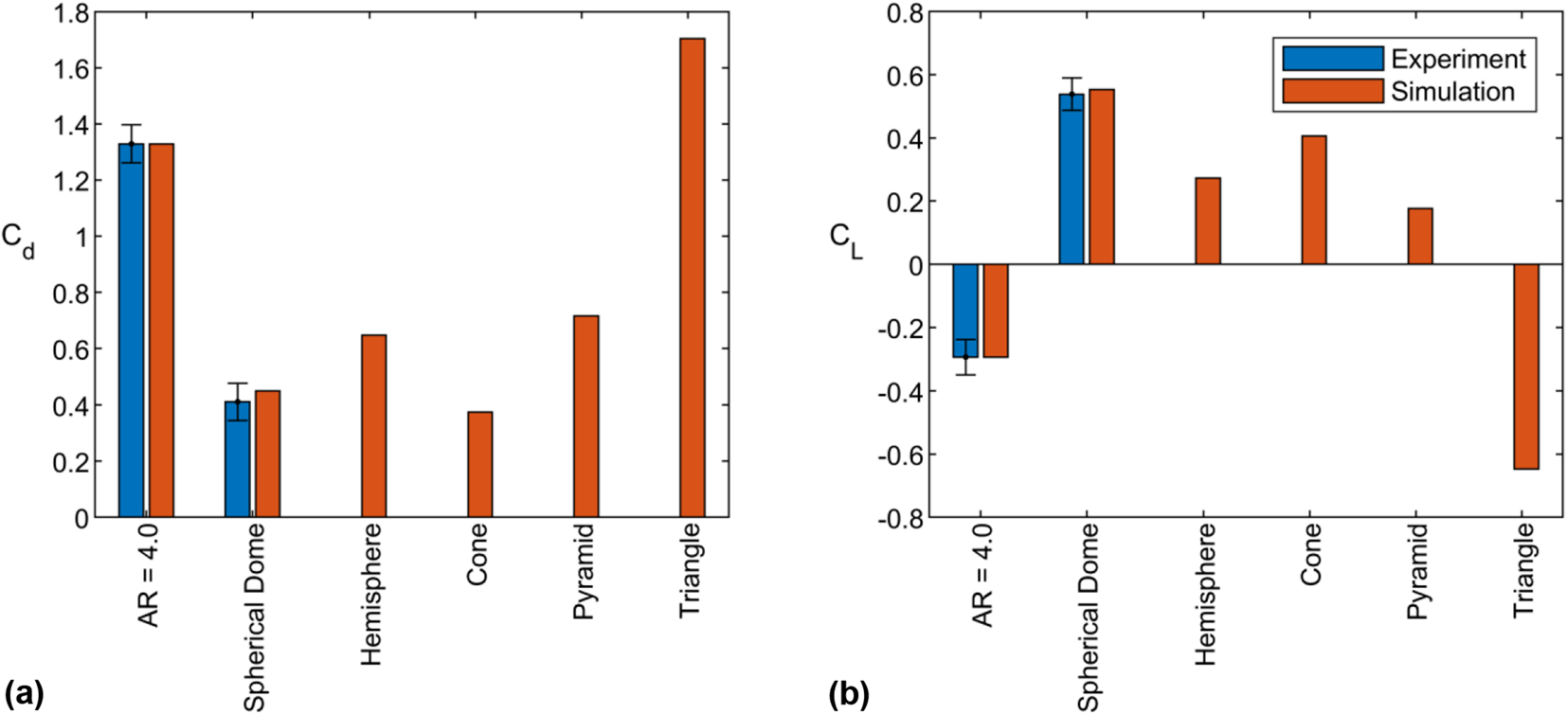
(**a**) Drag and (**b**) lift coefficients of models for experiments and simulations in a flow with speed 0.35 ms$^{-1}$.

As shown in Fig. 4, the drag and lift coefficients computed from the simulations agree, within uncertainty, with the values obtained in experiments for the $AR = 4.0$ sea star and spherical dome. Further, as shown in Fig. 3, the mean flow fields and wake structures obtained in the simulations are in good qualitative agreement with the PIV results. For instance, the vertical extent of the wake region is largest for the $AR = 4.0$ sea star and smallest for the spherical dome. These observations give us confidence that the CFD simulations can reasonably reproduce the flow physics observed in the real world experiments.

**Figure 5:**
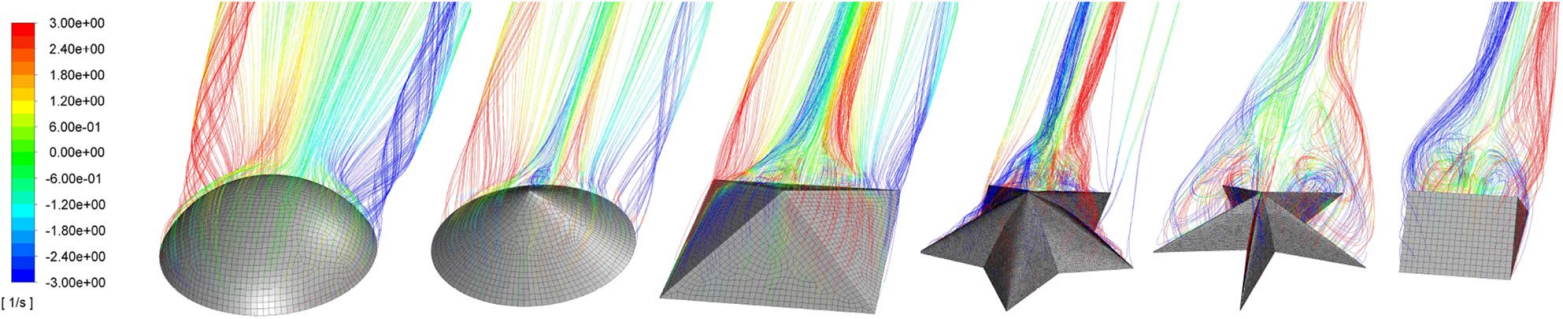
Pathlines of flow over a spherical dome, cone, pyramid, $AR=1.5$ sea star, $AR=4.0$ sea star, and triangular prism. The pathlines are colored based on the local streamwise vorticity. Objects are placed in order of descending lift force. Figure created using Ansys Fluent 2019 R2 https://www.ansys.com/.

The simulation results in Fig. 4b also suggest that downforce is not obtained for the pyramid or cone shapes, despite these objects having a sharp apex similar to the sea star models. Downforce is only observed for the triangular prism, and this downforce carries a significant drag penalty. These observations can be explained by considering the pathline visualizations shown in Fig. 5. For the downforce-producing shapes, the pathline visualizations show that the streamwise vorticity is negative (blue) on the left and positive (red) on the right side of the shapes. This results in a significant upwelling of fluid from the central region of the wake into the freestream. The momentum transport associated with this upwelling flow explains the high drag and negative lift generated by the sea star models and the triangular prism. Pathlines near the apex for the cone and pyramid shapes also show evidence of a similar arrangement in streamwise vorticity. However, pathlines at the base of the pyramid and cone shapes show a reversal in sign for the streamwise vorticity: the streamwise vorticity is positive (red) on the left and negative (blue) on the right, similar to the flow field observed around the spherical dome. This is indicative of a downwelling flow that transports high momentum fluid from the freestream into the wake and explains the lower drag and positive lift forces experienced by spherical dome, cone, and pyramid shapes. Note that the pathlines and vorticity distributions around the base of the lift-producing spherical dome, cone, and pyramid shapes in Fig. 5 are consistent with the horseshoe vortex systems typically observed in flows around surface mounted bodies^12,22,23^. Visualizations for the sea star bodies do not show evidence of this horseshoe vortex system.

## Discussion

The ability to stay attached to surfaces plays an important role in sea star locomotion and survival. The results presented in this study show that pentaradial sea star body shapes generate downforce independent of the incoming flow direction. This downforce could help sea stars avoid hydrodynamic dislodgement.

Hayne and Palmer^3^ showed that *Pisaster ochraceous* sea stars exhibit significant morphological plasticity in response to hydrodynamic conditions. Specifically, observations made in different environments showed a linear relationship between mean wave speed and sea star aspect ratio. Transplant studies confirmed this trend: sea stars moved into higher energy environments showed an increase in body aspect ratio. One hypothesis proposed to explain this correlation was that the change in body shape may enable sea stars to better resist hydrodynamic forces. Our results show that an increased aspect ratio produces a larger downforce, but this comes at the expense of a larger drag force. In the present study, sea star height and frontal area were maintained constant across the different aspect ratios tested. In contrast, the observations made by Hayne and Palmer^3^ indicate that the increase in sea star aspect ratio is also accompanied by a decrease in height, i.e., sea stars exhibit higher aspect ratios *and* reduced height in more energetic environments. It is possible that the high aspect ratio body shape generates greater downforce while the reduction in height limits the drag penalty.

Sea stars may also prioritize downforce maximization over drag minimization. Per Martinez^24^, the following condition, derived from a simple balance of moments, can be used to evaluate the possibility of animal detachment in steady flows:

1 $$\begin{aligned} \frac{(F_d)h}{(W-B-F_L)(L/2)} >1. \end{aligned}$$

Here $F_d$ is the drag force, *h* is the height of the center of mass, *W* is the weight of the organism, *B* is buoyancy, $F_L$ is the lift force, and *L* is the base length defined in Sect. 4. For a sea star of comparable size to the models tested here, $L \approx 19$ cm and $h \approx 5$ cm, the net vertical force, $W-B-F_L$, has approximately double the effect of the horizontal drag force, $F_d$. Thus, the higher downforce may still be beneficial for sea stars staying attached to surfaces despite the drag penalty incurred.

The preceding discussion suggests that the pentaradial body shape and morphological plasticity exhibited by sea stars enable them to better resist hydrodynamic loads. Sea urchins are often found in the same environment as sea stars and have a comparable biological adhesion mechanism^25–27^. Yet it is unlikely that the spiny spheroidal geometry typical of sea urchins leads to downforce generation. A characterization of the hydrodynamic forces acting on sea urchin body shapes may provide additional insight into how these organisms remain attached to surfaces in energetic flow conditions.

Although the ability to generate downforce has been observed previously for bilaterally symmetric aquatic organisms such as clams^28^ and crabs^24^, the orientation-independent nature of the downforce observed in this study is unique to the pentaradial sea star geometry. bilaterally symmetric organisms must either be passively aligned in the flow to generate downforce, as is the case for clams while swash-riding^28^, or actively posturing in the flow, as is the case with crabs in certain flow conditions^24^. In other words, bilaterally symmetric organisms require some degree of passive or active reorientation to produce downforce for different flow directions. On the other hand, sea star body shapes produce downforce independent of orientation relative to the flow. Since downforce is not produced by radially symmetric spherical domes and cones, we suggest that the pentaradial geometry of sea star bodies is unique in that it generates a hydrodynamic response that is similar to a (nearly) two-dimensional triangular prism but also insensitive to the incoming flow direction. Our observations suggest that the downforce generated by the sea star bodies and the triangular prism arises from the upwelling flow created along the centerline, with the breakdown of the horseshoe vortex system around the base also playing a role.

We recognize that the present work has some important limitations. Specifically, we only consider a limited range of (smooth) sea star morphologies in steady flow. Intertidal sea stars exhibit significant diversity in body shape, size, and surface texture^29^. Moreover, the intertidal zone is likely to be dominated by wave-driven unsteady flows in which inertial effects (e.g., added mass) can also play a role^1^. Nevertheless, this study presents the first evidence for downforce generation with pentaradial sea star body shapes. This orientation-independent downforce could have important implications for sea star locomotion and survival.

## Methods

### Flow facility and experiment setup

**Figure 6:**
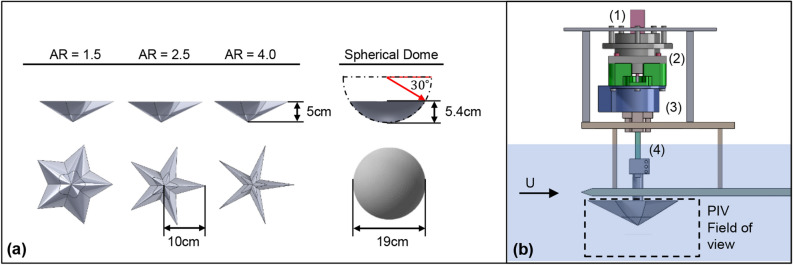
(**a**) Schematic showing the sea star and spherical dome models tested in the experiments. (**b**) Schematic of load cell-model attachment assembly, including: (1) servo for controlling orientation angle $\Theta$, (2) bearing and load cell coupling mount, (3) ATI Gamma load cell, (4) linear servo for vertical positioning.

All experiments were performed in a large-scale free surface water channel facility in the Fluid-Structure Interactions laboratory at USC. This facility has a glass-walled test section of length 7.6 m, width 0.9 m, and depth 0.6 m, and is capable of generating flows with freestream velocities up to $U \approx 0.6$ ms$^{-1}$. As shown in Fig. 6b, 3D-printed sea star and spherical dome models were mounted towards the leading edge a flat plate setup in the water channel. The models were mounted 3 cm from the leading edge of the plate to limit boundary layer development and positioned 5 mm from the plate surface. This distance was chosen to approximate the height of the tube feet (or podia) below the sea stars. Additional force measurements conducted with the models placed 25 mm from the plate surface showed very similar trends to the results presented in Sect. 2.1. A positioning system was used to precisely control model orientation $\Theta$ with respect to flow and vertical distance with respect to the smooth plate surface. An Arduino and a high torque Hitec servo motor controlled the rotation system. An Actuonix linear servo motor with 100 mm stroke controlled the vertical positioning. The models were tested in flows with freestream velocity ranging from roughly $U \approx 0.24$ ms$^{-1}$ to $U \approx 0.47$ ms$^{-1}$. A Laser Doppler Velocimeter (MSE miniLDV) placed 3 m downstream from the end of the flat plate setup was used to monitor the flow speed.

The models tested in the experiments were designed using SolidWorks and manufactured from polylactic acid (PLA) using a Prusa i3 3D-printer. Hydrodynamic forces on the models were measured using an ATI Gamma 6-axis load cell. A PIV system comprising a 5 W 532 nm continuous wave laser and a Phantom VEO high-speed camera was used for flow visualization and control volume analyses. All measurements were made with the models placed below the plate to eliminate free surface effects. A special faring was designed to isolate the positioning system above the flat plate from the flow, thereby ensuring that the forces measured originated from the models alone. Additional details are provided in the subsections below.

### 3D-printed models

The sea star models were created in SolidWorks by circular patterning an arm of specified aspect ratio around the central axis. Each arm profile is formed from a conic line of curvature $\gamma = 0.75$, length 10 cm and apex height 5 cm, as shown in Fig. 6. Three different sea star models were created by varying the arm width at the base, resulting in models with aspect ratios $AR = 4.0$, $AR = 2.5$, and $AR = 1.5$. Here, the aspect ratio is defined as the ratio between the arm length from distal tip to the central axis (10 cm) and the width at the base arm intersections.

The spherical dome used in this study is the top slice of a sphere with radius 11 cm. The slicing plane was placed at $30^\circ$ from the bisecting plane such that the base diameter for the dome was comparable to the frontal width of the sea star models, $L = 19$ cm, and the height of the dome was comparable to the apex height of the sea star, 5.4 cm. Frontal and planform areas for the models are shown in Table 2. All models were designed with a cylindrical clamp at the base that connected with the positioning system.

**Table 2:** Lengths and areas for experimental models. For the sea star models, $L = 19$ cm corresponds to the frontal width when one of the arms is oriented into the flow, $\Theta = 0^\circ$.

	$AR=4.0$	$AR=2.5$	$AR=1.5$	Spherical dome
Length, *L* [m]	0.19	0.19	0.19	0.19
Planform area, $A_p$ [$10^{-3}$ m$^2$]	8.9	9.7	16.1	28.4
Frontal area, $A_f$ [$10^{-3}$ m$^2$]	4.8	4.8	4.8	7.4

### Load cell measurements

The hydrodynamic drag and lift forces acting on the models were measured using an ATI Gamma 6-axis load cell capable of 0.00625N resolution in lateral forces and 0.0125 N resolution in vertical forces. Data from the load cell were logged to a PC using a 16-bit data acquisition system (National Instruments NI PCIe-6321). The sampling rate was set to 5kHz based on load cell manufacturer specifications. For each configuration, force data were collected for 60 s, yielding 300,000 samples. Prior to each measurement made in flow, a zero reading was collected to eliminate the effects of model weight, buoyancy, and load cell drift error from the measured hydrodynamic forces.

Following standard convention, the measured drag and lift forces were converted into drag and lift coefficients and expressed as a function of the Reynolds number. These dimensionless parameters were calculated using the following relations:

2 $$\begin{aligned} C_d&= \frac{2F_d}{\rho \ A_{f} \ U^2}, \end{aligned}$$

3 $$\begin{aligned} C_L&= \frac{2F_L}{\rho \ A_{p} \ U^2}, \end{aligned}$$

4 $$\begin{aligned} Re&= \frac{U \ L}{\nu }, \end{aligned}$$

where $\rho$ is fluid density, *U* is freestream velocity, $A_f$ is model frontal area, $A_p$ is model planform area, *L* is a characteristic length, and $\nu$ is the kinematic viscosity. The fluid density and kinematic viscosity were set to values expected for water at ambient temperature $20\,^\circ$C. Note that the drag coefficient is calculated using frontal area while the lift coefficient is calculated using the planform area.

### PIV and control volume analysis

The two-dimensional, two-component PIV system comprised a 5 W 532 nm continuous laser and a Phantom VEO 410 L high-speed camera fitted with a 50 mm f/1.4 Nikon lens. The camera recorded images at a rate of 400 Hz and the spatial resolution for the experiments was 0.23 mm per pixel. Because the laser sheet was not wide enough to illuminate both the fore and aft sections of the model, we fixed the position of the camera and moved the laser to obtain two sets of images that were then spliced together at the center of the object. Because of this splicing, we only report mean velocities and force estimates obtained after averaging over 997 frame-pairs. We used PIVLAB^30^ for background correction and subsequent PIV analyses. We used a multi-pass fast Fourier transform algorithm for the PIV analysis with a final interrogation window of size $32 \times 32$ pixels and 50% overlap.

The vector field data obtained from PIV were used to estimate lift and drag forces per unit length using a control volume approach. Specifically, for a fixed control volume and steady state conditions, the hydrodynamic force (${\mathbf {F}}$) can be estimated using the following relation

5 $$\begin{aligned} \int _{S} \rho ({\mathbf {u}} \ \cdot \ {\mathbf {n}}) {\mathbf {u}} \ dS = {\mathbf {F}}, \end{aligned}$$

in which *S* is the control surface bounding the control volume that encompasses the body, ${\mathbf {u}}$ is the velocity vector, ${\mathbf {n}}$ is the outward normal for the control surface, and ${\mathbf {F}}$ is the force imparted on fluid by the body. Though we do not have access to the full three-dimensional flow field from PIV, we can estimate drag and lift forces per unit length acting on the body ($F_d{^\prime }$, $F_L{^\prime }$) by considering a planar approximation to Eq. (5). Separating the momentum conservation relation into the streamwise (*x*) and wall-normal (*y*) directions, $F_d{^\prime }$ and $F_L{^\prime }$ can be estimated using:

6 $$\begin{aligned} \rho \left( \int _{H} u_{in}^2 dy - \int _W v_{top}u_{top} dx - \int _H u_{out}^2 dy \right) = F_d{^\prime } \end{aligned}$$

and

7 $$\begin{aligned} \rho \left( \int _H u_{in}v_{in} dy - \int _W v_{top}^2 dx - \int _H u_{out}v_{out} \mathbf {e_y} dy \right) = F_L{^\prime } \end{aligned}$$

where $u_{in}$ and $v_{in}$ are the streamwise and wall-normal velocities at the upstream (inlet) of the control area, $u_{out}$ and $v_{out}$ are the velocities at the downstream (outlet), and $u_{top}$ and $v_{top}$ are the velocities at the upper bounding surface. The height of the control area is *H* and the width is *W*.

### CFD simulation setup

ANSYS Fluent was used to simulate the flow field over the sea star and spherical dome models tested in the experiments, as well as several related geometries. These simulations were carried out using a coupled pressure-velocity method with a built-in steady-state $k-\epsilon$ turbulence model. The coefficients of the turbulence model were set to the default settings. For all models, the outer flow mesh was a coarse hex-dominant grid. For the near-field flow around the object and for three body-lengths downstream from the rear edge of the models, a fine tetrahedral mesh was used. This also ensured that the sharp geometries involved near the apex of the sea star, pyramid, cone, and triangular prism geometries were adequately resolved. Convergence tests indicated that meshes with roughly 800,000 elements provided a reasonable balance between accuracy and speed. For example, doubling the number of mesh elements beyond this value led to changes of $\le 4\%$ in the computed drag and lift forces for the highest aspect ratio sea star model.

For all the simulations, the working fluid was assumed to be water at 20 $^\circ$C. The models were set 5 mm from the bounding floor in the simulation and 3 cm the edge of the plate, consistent with the experiment setup shown in Fig. 6. The inlet velocity was set at $U = 0.35$ ms$^{-1}$, which is roughly the midpoint of the velocity range tested in the experiments.
